# Human Papilloma Viruses and Breast Cancer – Assessment of Causality

**DOI:** 10.3389/fonc.2016.00207

**Published:** 2016-09-29

**Authors:** James Sutherland Lawson, Wendy K. Glenn, Noel James Whitaker

**Affiliations:** ^1^School of BABS, University of New South Wales, Sydney, NSW, Australia

**Keywords:** breast cancer, human papilloma virus, HPV, causality

## Abstract

High risk human papilloma viruses (HPVs) may have a causal role in some breast cancers. Case–control studies, conducted in many different countries, consistently indicate that HPVs are more frequently present in breast cancers as compared to benign breast and normal breast controls (odds ratio 4.02). The assessment of causality of HPVs in breast cancer is difficult because (i) the HPV viral load is extremely low, (ii) HPV infections are common but HPV associated breast cancers are uncommon, and (iii) HPV infections may precede the development of breast and other cancers by years or even decades. Further, HPV oncogenesis can be indirect. Despite these difficulties, the emergence of new evidence has made the assessment of HPV causality, in breast cancer, a practical proposition. With one exception, the evidence meets all the conventional criteria for a causal role of HPVs in breast cancer. The exception is “specificity.” HPVs are ubiquitous, which is the exact opposite of specificity. An additional reservation is that the prevalence of breast cancer is not increased in immunocompromised patients as is the case with respect to HPV-associated cervical cancer. This indicates that HPVs may have an indirect causal influence in breast cancer. Based on the overall evidence, high-risk HPVs may have a causal role in some breast cancers.

## Introduction

High-risk human papilloma viruses (HPVs) may have a causal role in some breast cancers. However, the assessment of causality of HPVs in breast cancer is extremely difficult. The main difficulty is that HPV infections are common, but HPV-associated breast cancers are uncommon. Further, HPV infections may precede the development of breast and other cancers by years or even decades. Further, HPV oncogenesis can be indirect. Despite these difficulties, the emergence of new evidence has made this assessment a practical proposition.

A good starting point is to review the evidence according to the criteria developed by A. Bradford Hill in 1965 and later modified by others (1–3). The Hill criteria are (i) strength of the association, (ii) consistency, (iii) specificity, (iv) temporality (time sequence), (v) biological gradient, (vi) plausibility, (vii) coherence, (viii) experimental evidence, and (ix) analogy. There is an overlap between several criteria such as strength of the association and specificity. If viruses, such as high risk for cancer HPVs, fulfill all or most of these criteria, there is a likely relationship, but not necessarily a conclusive confirmation of causality. One weakness of the Hill criteria is that each is given similar weight or importance. This is in contrast to the key requirement of zur Hausen that there should be a regular presence of the nucleic acid of the virus in specific malignant tumors (3). Even this criteria may be difficult because of the ability of HPVs to act indirectly by causing genetic instability or promoting the influence of other viruses such as Epstein Barr virus (EBV).

### Strength of the Association between Identification of HPV and Breast Cancer

The HPV viral load in breast cancer is extremely low as compared to HPV in cervical cancer (approximately 2,000-fold lower, see Table 1) (4, 5). As a consequence, identification of HPV in breast tumors is difficult whether by amplification techniques such as polymerase chain reaction (PCR) or massive parallel sequencing. The low viral load is the likely reason that several laboratories have not been able to detect HPV. On the other hand, HPV gene sequences have been identified in breast tumors in over 40 studies conducted in 20 countries (6, 7).

**Table 1:** **HPV viral loads in HeLa (cervical cancer cell line), breast cancer tissues, and cervical cancer tissues (4, 5)**.

	HeLa cells	Breast cancer	Cervical cancer
HPV gene copy/cell	10–50	0.00054–0.0021	13
HPV gene copy/ng	1,510–7,558	0.082–0.32	2,000
HPV gene copy/50 ng	75,500–377,000	4–16	98,500

*The viral load is approximately 2,000-fold greater in cervical cancer as compared to breast cancer*.

*ng = nanogram*.

**Figure 1:**
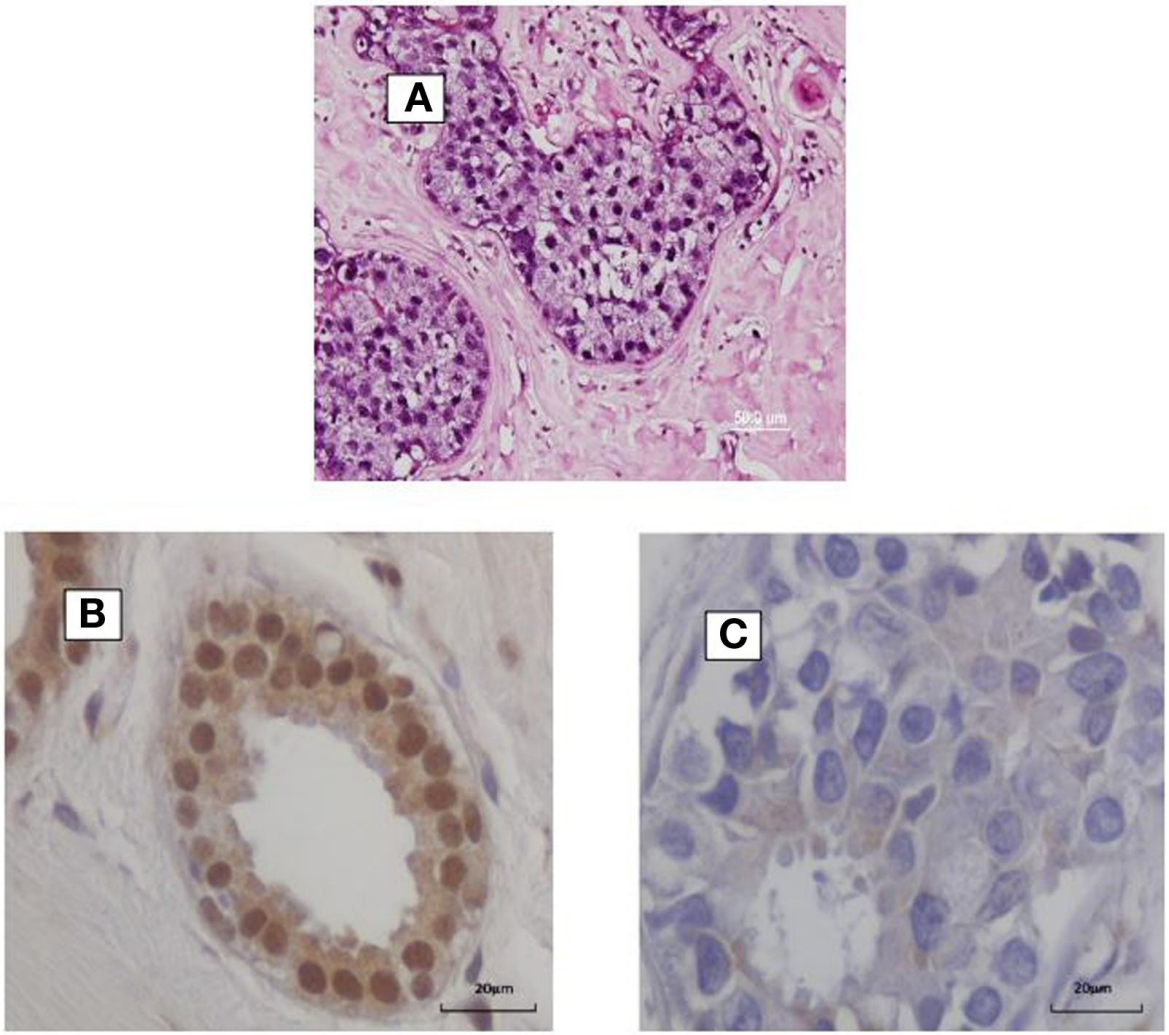
**(A)** HPV identified by *in situ* PCR in breast ductal carcinoma in situ. Koilocytes are present in this specimen. **(B,C)** HPV E7 protein in a benign breast specimen **(B)**. No HPV E7 protein in invasive breast cancer 4 years later in the same patient **(C)**.

The prevalence of high-risk HPV positive breast cancers varies between 0 and 2% in some Chinese Provinces to 86% in North America (7). HPV types 16 and 18 are the most prevalent, but HPV 33 and 58 are common in China and Japan (8, 9). A recent meta-analysis of 22 case–control studies indicated that HPV infection was associated with an increased risk of breast cancer as compared to benign or normal breast controls (odds ratio 4.02) (7). Increased risk of breast cancer is influenced by geographic region, PCR techniques, and publication period. All of these studies were based on PCR analyses. HPV gene sequences identified by *in situ* PCR are shown in Figure 1. High risk HPVs have been identified in invasive breast cancers using Next Generation Sequencing (NGS), but this technique is much less sensitive than PCR (10).

Case–control studies, which compare the prevalence of high-risk HPVs in breast cancer as compared to benign breast or normal breast are shown in Table 2. The prevalence of HPV is fourfold higher in breast cancer (21.5%) than controls (5.1%) in studies conducted in different laboratories in a wide range of countries. This parallels the findings of a recent meta-analysis based on selected studies, which are included in this Table (7). It is of interest that high-risk HPVs were identified in only 4.1% of benign or normal (usually from cosmetic surgery) as compared to 17.7% in non-cancerous breast tissues located next to breast cancer tissues. The implication is that HPVs may be have increased prevalence in non-cancerous breast tissues located next to cancerous breast tissues and should not be used as controls.

**Table 2:** **Identification of high-risk HPVs in breast cancer and benign or normal breast controls in case–control studies**.

Reference	Country	HPV-positive breast cancers/total breast cancers	HPV-positive non-cancer breast/total non-cancer breast controls	Main HPV types
Yu et al. (8)	Japan/China	18/52 (35%)	0/15 (0%)	18, 33
Damin et al. (11)	Brazil	25/101 (25%)	0/41 (0%)	16, 18
Tsai et al. (12)	Taiwan	8/62 (13%)	2/42 (5%)	Not available
Choi et al. (13)	Korea	8/123 (7%)	0/31 (0%)	16, 18, 58
Gumus et al. (14)	Turkey	37/50 (74%)	9/16 (56%)	18, 33
He et al. (15)	China	24/40 (60%)	1/20 (5%)	16
de Leon et al. (16)	Mexico	15/41 (37%)	0/43 (0%)	16, 18
Heng et al. (17)	Australia	8/26 (31%)	3/28 (11%)	16, 18
Herrera-Romano et al. (5)	Mexico	6/60 (10%)	7/60 (12%)	16
Mou et al. (18)	China	4/62 (6%)	0/46 (0%)	16, 18
Chang et al. (19)	China	0/48 (0%)	3/30 (10%)	6, 11
Sigaroodi et al. (20)	Iran	15/43 (35%)	1/40 (3%)	16, 18
Frega et al. (21)	Italy	9/31 (29%)	0/12 (0%)	16, 18
Glenn et al. (22)	Australia	25/50 (50%)	8/40 (20%)	16, 18
Liang et al. (23)	China	48/224 (21%)	6/37 (16%)	16, 18, 33, 58
Ahangar-Oskouee et al. (24)	Iran	22/65 (34%)	0/65 (0%)	16
Ali et al. (25)	Iraq	60/129 (47%)	3/41 (7%)	16, 18, 33
Eslamifar et al. (26)	Iran	0/100 (0%)	0/50 (0%)	
Manzouri et al. (27)	Iran	10/55 (18%)	7/51 (14%)	16
Peng et al. (28)	China	2/100 (2%)	0/50 (0%)	18
Fu et al. (9)	China	25/169 (15%)	1/83 (1%)	58
Li et al. (29)	China	3/187 (2%)	0/92 (0%)	6, 16, 18
Zhou et al. (6)	China	0/77 (0%)	0/77 (0%)	
Vernet-Tomas et al. (30)	Spain	0/76 (0%)	0/2 (0%)	

*These data indicate that high-risk HPVs are fourfold more prevalent in breast tumors as compared to non-cancer controls. This parallels the findings of a recent meta-analysis based on selected studies included in this Table (7)*.

In a recent study, it has been shown that women with HPV-associated cervical pathology are at increased risk from the same HPV type positive breast cancer (31). This observation confirms large epidemiological studies, which show that women with squamous or glandular precancer of the cervix have a significantly higher risk of subsequent breast cancer than the general female population – odds ratios 1.10 and 1.52, respectively (32, 33).

### Consistency

The HPV viral load in breast cancer is extremely low (see Table 1). Accordingly, the identification of HPVs by both PCR and NGS is difficult. This may explain the very different findings from the 10 case–control studies conducted in various locations in China. The identification of HPVs in China vary from 0 to 60%. Similarly, the findings in Iran vary from 0 to 34%. There are three relevant studies based on NGS – Tang et al., Khoury et al., and Lawson et al. (10, 34, 35). High risk HPVs were not identified in breast cancers from the The Cancer Genome Atlas (TCGA) series of approximately 800 breast cancers in either the Tang or Khoury studies but were identified in 2.3% of the same series by Lawson et al. (10, 34, 35). Clearly, PCR-based analyses are much more sensitive than current NGS-based analyses. The reasons for this discrepancy have been considered by Vinner et al. who has shown that NGS-based techniques are unlikely to detect viruses with very low loads in cancers as compared to amplification techniques such as PCR (36).

Despite these differences, the outcome of the PCR-based case–control studies is consistent despite having been conducted with different techniques, locations, and times by different research groups (Table 2). As stated above, the prevalence of high-risk HPVs in breast cancers is fourfold higher than in normal and benign breast controls. This is a substantial difference between benign breast and breast cancers and cannot be attributed to differences in laboratory techniques.

### Epidemiology

If HPV infections were major contributors to breast cancer, it would be anticipated that countries with high rates of HPV associated cervical cancer would also have high rates of HPV associated breast cancer. The opposite appears likely, namely that countries with low rates of cervical cancer appear to have high rates of breast cancer (37). This is shown in Table 3. While these data must be considered only as broad trends, the implication is that HPVs are unlikely to be major contributors to breast cancer. However, it remains possible that HPVs may be involved in some but not all breast cancers.

**Table 3:** **Incidence and mortality per 100,000 women (age adjusted) of cervical and breast cancers**.

	Incidence cervical cancer	Incidence breast cancer	Mortality cervical cancer	Mortality breast cancer
France	8	92	2	17
England	7	90	2	18
United States	5	84	4	14
Australia	5	82	2	14
Canada	5	76	3	15
China	6	44	5	5
India	16	32	10	10
Japan	7	32	4	8
Thailand	23	25	10	10

*Selected high and low cervical cancer country data (17)*.

*Low cervical cancer correlates with higher breast cancer incidence and mortality (incidence *p* = 0.052, mortality *p* = 0.118). As HPV is almost universally associated with cervical cancer, these data suggest that HPV is not a major cause of breast cancer. These data demonstrate trends only. Incidence is influenced by screening, and mortality is influenced by accuracy of diagnosis and effectiveness of treatment*.

Epidemiological combined with experimental data have demonstrated that (i) the prevalence of high-risk HPVs is high in early as compared to late breast cancer, (ii) this early influence of HPVs is indirect and operates by the destabilizing effects of HPV on the human genome *via* APOBEC3B enzyme (38). This influence of HPV on APOBEC enzymes has been confirmed (39). It has also been demonstrated that high expression of HPV E7 protein in benign breast tissues as compared to low HPV E7 expression in subsequent HPV positive breast cancer occurs in the same patients (40). This is additional evidence that the influence of HPVs occurs early in breast cancer.

This indirect influence of HPVs in breast cancer needs to be considered in the context of the epidemiological observations by Grulich and Vajdic (41) that the prevalence of breast cancer is not increased in immunocompromised patients (patients with HIV infections or organ transplant recipients). This compares to the fourfold to sixfold increase in cervical cancer in these patients. Again the implication is that the influences of HPV are indirect and occur early in breast oncogenesis.

There is also evidence that HPVs may collaborate with EBV and which together may have oncogenic influences. The co-prevalence of HPVs and EBV in nasopharyngeal and breast cancers has been demonstrated (22, 42, 43).

### Specificity

Specificity is a very weak causative criterion in both communicable and non-communicable diseases and probably should not be included in the Hill list of causal criteria. Any associations between HPVs and breast cancer are obviously not specific. HPV is well established as a causal factor in other cancers such as cervical and head and neck cancers. In addition, high-risk HPVs have been identified in a wide range of other cancers and non-cancerous tissues (44). In the past, HPVs have been regarded as being specific to epithelial cells including breast epithelial cells. However, HPV gene sequences have been identified in non-epithelial cell brain tumors and in coronary and aorta artery smooth muscle cells (45).

### Temporality (Time Sequence)

High-risk HPVs have been identified in benign breast tissues of the same type as in breast tumors, which subsequently developed in the same patients (10). The identification of HPVs preceded the development of breast cancer in the same patients by between 1 and 11 years. As this study was retrospective, the time of the original HPV infection cannot be determined. Similarly, HPV-associated cervical infections and abnormal cervical pathology can precede the development of same type HPV positive breast cancer in the same patient (38, 46). Again, however, the time of the original HPV infection cannot be determined.

These observations confirm that the HPV infections precede the development of HPV-positive breast cancer.

### Biological Gradient, Plausibility, and Coherence

These criteria overlap. The biological gradient or response differs greatly with respect to the HPV viral load in breast as compared to cervical cancer. The HPV viral load in breast cancer is 2,000-fold less than in cervical cancer, as shown in Table 1. This viral load is so low that any direct HPV oncogenic influence is likely to be small. It is possible that HPV oncogenic influences in breast cancer are indirect. This is because there is no increase in the prevalence of breast cancer in patients with induced immunosuppression or with immunity suppressed by human immunodeficiency virus (HIV) (41). This is in contrast to the up to sixfold increase in prevalence of HPV associated cervical cancer in similar patients. Transcription from HPV DNA to RNA in HPV-associated breast cancer has not been identified by some investigators (47). However, a very low prevalence of HPV transcripts has been identified in the TCGA series of breast tumors (10). An indication of some HPV viral transcription in some breast cancers is the identification of HPV E7 protein expression (10).

The oncogenic capacity of high-risk HPVs is well established (3). Accordingly, it is plausible that high-risk HPVs could have a role in breast cancer. The transmission of HPVs during sexual activities involving surface to surface contact between epithelial cells is also well documented (48). Not so well known is the identification of high-risk HPVs in white blood cells, which suggests a means of HPV transmission throughout the body (49).

Band et al. have repeatedly demonstrated that HPV DNAs can immortalize normal human mammary epithelial cells in experimental conditions (50, 51). This evidence demonstrates that HPVs have the capacity to have a causal role in breast cancer. However, it is not conclusive evidence of causality because the influence of HPVs and their oncogenic proteins may differ in experimental conditions than in normal women.

### Mechanisms of Oncogenesis

The oncogenic mechanisms by which HPV induces cervical cancer have been intensively studied (3). High-risk HPV encodes a series of proteins, designated as early (E1–E7) or late (L1 and L2). Although all of the viral proteins have a role in viral replication, only a small number of the viral early proteins have a role in cellular transformation. Transformation is due to the HPV E6 and E7 oncoproteins, which work in concert to disrupt cell-cycle regulation, inhibit apoptosis, and stimulate cell cycle progression by binding/inhibiting the p53 and p110RB tumor suppressor genes, respectively.

p53 protein expression has been shown to be significantly inhibited in HPV-positive breast cancer as compared to HPV-negative breast cancer (46). This indicates that HPVs in breast cancer may be biologically active.

In addition, HPV E5 and E6 act early in transformation (before integration) and can disrupt cytokeratin causing perinuclear cytoplasmic clearing and nuclear enlargement, which leads to the appearance of a koilocyte (52). Koilocytes are shown in Figure 1. HPV associated koilocytes have been identified in breast tumors, which is an indication of early influences of HPVs on normal breast epithelial cells (53). Oncogenic protein HPV E7 expression has been observed in both benign and subsequent breast cancers in the same patients (10). This is an indication of HPV oncogenic activity. HPV type 16 E6 and E7 oncoproteins have been shown to convert non-invasive breast cancer cells into an invasive form both *in vitro* and in nude mice (54).

HPV E7 is more frequently expressed in benign breast biopsy specimens than in subsequent breast cancers that developed several years after the biopsy (40). This is shown in Figure 1. This suggests that HPVs may have an early role in breast cancer – the “hit and run” phenomenon. These direct influences are probably limited because, as indicated above, there is no increase in the prevalence of breast cancer in immunocompromised patients (41). If HPVs had a major direct influence in breast cancer, it would be anticipated that the prevalence would be increased in these patients.

Recent evidence suggests that high-risk HPVs may have an indirect oncogenic influence in the development of breast cancer. HPVs can promote the activity or influences of a DNA-editing enzyme APOBEC3B, which is a protein encoded by the APOBEC3B gene (38, 39). Upregulated APOBEC3B protein expression was first identified by Burns et al. as an enzymatic source of mutations in breast cancer (55). Upregulation of APOBEC3B can occur in many different cancers (55). It has also been shown that high expression of APOBEC3B can be associated with aggressive breast cancers (56).

Finally, HPVs may collaborate with other oncogenic viruses such as EBV to cause breast cancer. The evidence for such a collaboration is very limited and mainly consists as an association (22, 43, 57). There is unpublished experimental evidence, which supports this notion (58).

## Discussion and Conclusion

The fourfold higher prevalence of high-risk HPVs in breast cancer as compared to normal and benign breast tissues is a consistent finding among over 25 studies conducted in different laboratories in many contrasting populations. This phenomenon is strongly suggestive of a role of high-risk HPVs in breast cancer. However, the nature of this role is far from clear. HPVs do not appear to have a direct oncogenic influence in breast cancer as demonstrated by the fact that the prevalence of breast cancer is not increased in immunocompromised patients (as compared to the fourfold to sixfold increase in cervical cancer in these patients). HPV E7 oncprotein is highly expressed in benign breast tissues but lowly expressed in breast cancer tissues in the same patients. This is an indication of early HPV influences. The implication is that the influences of HPV in breast cancer are probably early and indirect and without major influences of the immune system.

It can be argued that ubiquitous infections such as with HPVs cannot be associated with cancer. However, this is not a correct argument as shown by the example of EBV. Over 90% of Western populations become infected to EBV at some time in their lives. Rarely, these ubiquitous EBV infections cause serious cancers such as Burkitt’s lymphoma, nasopharyngeal cancer, and lymphomas. Interestingly, EBV infections that lead to cancer frequently do so in collaboration with other infectious agents, such as malaria, in the case of Burkitt’s lymphoma and HPVs in the case of nasopharyngeal cancers.

With the exception of the unimportant criteria of specificity, all the criteria for a causal role for high-risk HPVs in breast cancer have been met. High-risk HPVs have a well-documented oncogenic capacity (3). HPVs have been consistently identified in breast cancers by different workers in different laboratories in a wide range of countries (7). The odds ratio of HPV positive breast cancers is high as compared to benign breast biopsy and normal breast controls (7). There is evidence of HPV breast infection prior to the development of HPV positive breast cancer (10, 31, 38). HPV-associated cervical pathology can precede same HPV type positive breast cancer, and women with cervical pathology are at greater than expected risk of developing breast cancer (31, 46). There are plausible mechanisms, which indicate that HPVs may have an oncogenic influence on the breast (38).

However, the extremely low HPV viral load in breast cancers and the unchanged prevalence of breast cancer in immunocompromised women indicate a need for cautious conclusions.

Also, because multiple viruses (mouse mammary tumor virus, EBV, and bovine leukemia virus) may have roles in human breast cancer, it will be difficult to identify the influence of a vaccine aimed at a single virus. On the other hand, given the strong possibility that high-risk HPVs do have a role in some breast cancers, it is reasonable to promote the use of HPV vaccines, such as Gardasil, as they are very effective in reducing the risk of cervical cancer.

Based on this evidence, high-risk HPVs may have a causal role in a subset of breast cancers.

## Author Contributions

JL, WL, and NW – concepts and preparation of manuscript.

## Conflict of Interest Statement

The authors declare that the research was conducted in the absence of any commercial or financial relationships that could be construed as a potential conflict of interest. The reviewer AC and handling Editor declared their shared affiliation, and the handling Editor states that the process nevertheless met the standards of a fair and objective review.
